# A formal proof and simple explanation of the QuickXplain algorithm

**DOI:** 10.1007/s10462-022-10149-w

**Published:** 2022-04-07

**Authors:** Patrick Rodler

**Affiliations:** University of Klagenfurt: Alpen-Adria-Universitat Klagenfurt, Universitätsstr. 65-67, 9020 Klagenfurt, Austria

**Keywords:** QuickXplain, Correctness Proof, Proof to Explain, Algorithm, Find Irreducible Subset with Monotone Property, Minimal Unsatisfiable Subset, Minimal Correction Subset, Model-Based Diagnosis, Constraint Satisfaction Problem (CSP), Minimal Set Subject to a Monotone Predicate (MSMP), Relaxation of Overconstrained Problems, Conflict Computation, Ontology Debugging and Repair, Computation of Justifications

## Abstract

In his seminal paper of 2004, Ulrich Junker proposed the QuickXplain algorithm, which provides a divide-and-conquer computation strategy to find within a given set an irreducible subset with a particular (monotone) property. Beside its original application in the domain of constraint satisfaction problems, the algorithm has since then found widespread adoption in areas as different as model-based diagnosis, recommender systems, verification, or the Semantic Web. This popularity is due to the frequent occurrence of the problem of finding irreducible subsets on the one hand, and to QuickXplain’s general applicability and favorable computational complexity on the other hand. However, although (we regularly experience) people are having a hard time understanding QuickXplain and seeing why it works correctly, a proof of correctness of the algorithm has never been published. This is what we account for in this work, by explaining QuickXplain in a novel tried and tested way and by presenting an intelligible formal proof of it. Apart from showing the correctness of the algorithm and excluding the later detection of errors (*proof and trust effect*), the added value of the availability of a formal proof is, e.g., *(i)* that the workings of the algorithm often become completely clear only after studying, verifying and comprehending the proof (*didactic effect*), *(ii)* that the shown proof methodology can be used as a guidance for proving other recursive algorithms (*transfer effect*), and *(iii)* the possibility of providing “gapless” correctness proofs of systems that rely on (results computed by) QuickXplain, such as numerous model-based debuggers (*completeness effect*).

## Introduction

The task of finding within a given universe an irreducible subset with a specific monotone property is referred to as the *MSMP* (*M*inimal *S*et subject to a *M*onotone *P*redicate) *problem* (Marques-Silva et al. 2007, 2013). Take the set of clauses $S:=\{\lnot C, A\vee \lnot B, C\vee \lnot B, \lnot A, B \}$ as an example. This set is obviously unsatisfiable. One task of interest expressible as an MSMP problem is to find a minimal unsatisfiable subset (MUS) of these clauses (which can help, e.g., to understand the cause of the clauses’ inconsistency). At this, *S* is the *universe*, and the predicate that tells whether a given set of clauses is satisfiable is *monotone*, i.e., any superset (subset) of an unsatisfiable (satisfiable) clause set is unsatisfiable (satisfiable). In fact, there are two MUSes for *S*, i.e., $\{\lnot C, C\vee \lnot B, B\}$ and $\{A\vee \lnot B, \lnot A, B\}$. We call a task, such as MUS, that can be formulated as an MSMP problem a *manifestation of the MSMP problem*.

MSMP is relevant to a wide range of computer science disciplines, including model-based diagnosis (Jannach and Schmitz 2016; Rodler 2015; Kalyanpur 2006; Rodler and Herold 2018), constraint satisfaction problems (Junker 2001, 2004; Lecoutre et al. 2006), verification (Bradley and Manna 2007, 2008; Nadel 2010; Andraus et al. 2008), configuration problems (Felfernig et al. 2004; White et al. 2010), knowledge representation and reasoning (Darwiche 2001; McCarthy 1980; Eiter et al. 2009; Marquis 1995), recommender systems (Felfernig et al. 2006, 2009), knowledge integration (Rodler et al. 2013; Meilicke 2011), as well as Description Logics and the Semantic Web (Kalyanpur 2006; Rodler et al. 2019; Shchekotykhin et al. 2012; Horridge 2011; Schlobach et al. 2007; Schekotihin et al. 2018). In all these fields, (sub)problems are addressed which are manifestations of the MSMP problem. Example problems—most of them related to the Boolean satisfiability problem—are the computation of *minimal unsatisfiable subsets* (Marques-Silva et al. 2013; Dershowitz et al. 2006; Oh et al. 2004; Liffiton and Sakallah 2008) (also termed *conflicts* (Reiter 1987; de Kleer and Williams 1987) or *minimal unsatisfiable cores* (Dershowitz et al. 2006)), *minimal correction subsets* (Birnbaum and Lozinskii 2003; Marques-Silva et al. 2013; Rodler 2020) (also termed *diagnoses* (Reiter 1987; de Kleer and Williams 1987)), *prime implicants* (Slagle et al. 1970; Quine 1959) (also termed *justifications* (Horridge 2011)), *prime implicates* (Marquis 1995; Manquinho et al. 1997; Déharbe et al. 2013), and *most concise optimal queries to an oracle* (Rodler et al. 2013; Schekotihin et al. 2018; Rodler 2016; Rodler et al. 2017).

Numerous algorithms to solve manifestations of the MSMP problem have been suggested in literature, e.g., Marques-Silva et al. (2013), Rodler (2015), Junker (2001), Junker (2004), Bradley and Manna (2007), Bradley and Manna (2008), Rodler et al. (2017), Shchekotykhin et al. (2014), Shchekotykhin et al. (2015), Felfernig et al. (2012), Belov and Marques-Silva (2012). For instance, the algorithm proposed by Felfernig et al. (2012) addresses the problem of the computation of minimal correction subsets (diagnoses), and the one suggested by Rodler et al. (2017) computes minimal oracle queries that preserve some optimality property. In general, an algorithm *A* for a specific manifestation of the MSMP problem can be used to solve arbitrary manifestations of the MSMP problem if *(i)* the procedure used by *A* to decide the monotone predicate is used as a black-box (i.e., given a subset of the universe as input, the procedure outputs 1 if the predicate is true for the subset and 0 otherwise; no more and no less), and *(ii)* no assumptions or additional techniques are used in *A* which are specific to one particular manifestation of the MSMP problem.

Not all algorithms meet these two criteria. For instance, there are algorithms that rely on additional outputs beyond the mere evaluation of the predicate (e.g., certificate-refinement-based algorithms (Marques-Silva et al. 2013)), or glass-box approaches that use non-trivial modifications of the predicate decision procedure to solve the MSMP problem (e.g., theorem provers that record the axioms taking part in the deduction of a contradiction while performing a consistency check (Kalyanpur 2006)). These methods violate (i). Moreover, e.g., algorithms geared to the computation of minimal unsatisfiable subsets that leverage a technique called model rotation (Marques-Silva and Lynce 2011) are not applicable, e.g., to the problem of finding minimal correction subsets, since there is no concept equivalent to model rotation for minimal correction subsets (Marques-Silva et al. 2013). Thus, such algorithms violate (ii).

Among the general MSMP algorithms that satisfy (i) and (ii), QuickXplain (Junker 2004) (${\textsc {QX}}$ for short), proposed by Ulrich Junker in 2004, is one of the most popular and most frequently adopted.^1^ Likely reasons for the widespread use of ${\textsc {QX}}$ are its mild theoretical complexity in terms of the number of (usually expensive^2^) predicate evaluations required (Marques-Silva et al. 2013; Junker 2004), as well as its favorable practical performance for important problems (such as conflict (Shchekotykhin et al. 2008) or diagnosis (Shchekotykhin et al. 2014) computation for model-based diagnosis). In literature, ${\textsc {QX}}$ is utilized in different ways; it is *(a)* *(re)used as is* for suitable manifestations of MSMP (Felfernig et al. 2004), *(b)* *adapted* in order to solve other manifestations of MSMP (Rodler 2015), as well as *(c)* *modified or extended*, respectively, e.g., to achieve a better performance for a particular MSMP manifestation (Marques-Silva et al. 2013), to solve extensions of the MSMP problem (Rodler et al. 2017), or to compute multiple minimal subsets of the universe in a single run (Shchekotykhin et al. 2015).

Despite its popularity and common use, from the author’s experience and a recently conducted structured survey^3^, ${\textsc {QX}}$ appears to be quite poorly understood by reading and thinking through the algorithm, and, for most people, requires significant and time-consuming attention until they are able to properly explain the algorithm. In particular, people often complain they do not see why it correctly computes a minimal subset of the universe. This is not least because no proof of ${\textsc {QX}}$ has yet been published.

In this work, we account for this by *(1)* explaining ${\textsc {QX}}$ by means of an alternative tried and tested “flat” notation that proved to convey the intuition behind the algorithm well and to be more accessible to people than the usually adopted tree notation in our experience, and by *(2)* presenting a clear and intelligible proof of ${\textsc {QX}}$. The public availability of a proof comes with several benefits and serves i.a. the following purposes:

- *Proof Effect* *(a)* It shows ${\textsc {QX}}$’s correctness and makes it verifiable for everyone in a straightforward step-by-step manner (without the need to accomplish the non-trivial task of coming up with an own proof). *(b)* It creates compliance with common scientific practice. That is, every proposal of an algorithm should be accompanied with a (full and public) formal proof of correctness. This demand is even more vital for a highly influential algorithm like ${\textsc {QX}}$.
- *Didactic Effect* *(a)* It promotes (proper and full) understanding (Hanna and Jahnke 1996) of the workings of ${\textsc {QX}}$, which is otherwise for many people only possible in a laborious way (e.g., by noting down and exercising through examples and attempting to verify ${\textsc {QX}}$’s soundness on concrete cases). *(b)* It provides the basis for understanding (hundreds of) other works or algorithms that use, rely on, adapt, modify or extend ${\textsc {QX}}$.
- *Completeness Effect* It is necessary to establish and prove the full correctness of other algorithms that rely on (the correctness of) ${\textsc {QX}}$, such as a myriad of algorithms in the field of model-based diagnosis.
- *Trust and Sustainability Effect* It excludes the possibility of the (later) detection of flaws in the algorithm, and is thus the only basis for placing full confidence in the proper-functioning of ${\textsc {QX}}$.^4^
- *Transfer Effect* It showcases a proof template for recursive algorithms and may thus provide guidance to researchers when approaching the (often challenging task of formulating a) proof of other recursive algorithms.

The rest of this paper is organized as follows. We discuss related work in Sect. 2, before we briefly introduce the theoretical concepts required for the understanding and proof of ${\textsc {QX}}$ in Sect. 3. Then, in Sect. 4, we state the ${\textsc {QX}}$ algorithm in a (slightly) more general formulation than originally published in Junker (2004), i.e., we present ${\textsc {QX}}$ as a general method to tackle the MSMP problem.^5^ In addition, we explain the functioning of ${\textsc {QX}}$, and present an illustrative example using a notation that proved particularly comprehensible in our experience.^6^ The proof is given in Sect. 5. In Sect. 6 we explain the proof template we adopted in our proof, which may serve as a reference point for proving other recursive algorithms as well. Concluding remarks are made in Sect. 7.

## Related work

Bradley and Manna (2007, 2008) discuss and prove an MSMP algorithm dubbed min. As a side note in Bradley and Manna (2008), they mention that min is equivalent to an algorithm also called QuickXplain which was proposed earlier in Junker (2001) (we refer to this latter algorithm by ${\textsc {QX}}_{\mathsf {old}}$). Apart from the fact that there is no proof that min is indeed equivalent to ${\textsc {QX}}_{\mathsf {old}}$ (which is not clear from the descriptions in Bradley and Manna (2007, 2008)), both of these algorithms, min and ${\textsc {QX}}_{\mathsf {old}}$, are different to ${\textsc {QX}}$.

First, ${\textsc {QX}}_{\mathsf {old}}$, although returning the same output, is not equal to ${\textsc {QX}}$. A critical difference, e.g., is the algorithm part consisting of lines 5–11 in ${\textsc {QX}}_{\mathsf {old}}$ (Junker 2001, Fig. 3), which is not used in ${\textsc {QX}}$ and which affects the algorithm’s complexity. Also, note that no public proof is available for ${\textsc {QX}}_{\mathsf {old}}$. Second, min, although being similar to ${\textsc {QX}}$ and having the same worst-case complexity given that ${\textsc {QX}}$ uses a divide-and-conquer strategy that splits sets into equally-sized subsets, works in fact differently from ${\textsc {QX}}$. To see this, consider a universe of elements $\{1,2,3,4,5,6\}$ which subsumes exactly the two minimal subsets $X_1=\{1,2,4\}$ and $X_2=\{4,6\}$ subject to a monotone predicate *p*. In this case, min would return $X_2$, whereas ${\textsc {QX}}$ would output $X_1$. Table 1 summarizes the discussed differences between our work and related ones (Junker 2001, 2004; Bradley and Manna 2007, 2008).

**Table 1:** Comparison of the present work with related works wrt. the discussed algorithm and proof. By $\textsc {min}$ we refer to the algorithm stated in (Bradley and Manna 2007, Fig. 2) as well as in (Bradley and Manna 2008, Fig. 3), by ${\textsc {QX}}_{\mathsf {old}}$ to the algorithm also named QuickXplain given in (Junker 2001, Fig. 3), and by $\textsc {QX}$ to the algorithm given in (Junker 2004, Fig. 1). The $\checkmark ^{(*)}$ holds under the condition that the split function (which rules the divide-and-conquer mechanism, cf. Sect. 4) used in $\textsc {QX}$ is defined to partition sets into equally-sized subsets

	Alg. 1 (this work)	$\textsc {min}$	${\textsc {QX}}_{\mathsf {old}}$	${\textsc {QX}}$
Equivalence to ${\textsc {QX}}$	$\checkmark$	$\times$	$\times$	$\checkmark$
Same complexity as ${\textsc {QX}}$	$\checkmark$	$\checkmark ^{(*)}$	$\times$	$\checkmark$
Same output as ${\textsc {QX}}$	$\checkmark$	$\times$	$\checkmark$	$\checkmark$
Correctness proof given	$\checkmark$	$\checkmark$	$\times$	$\times$

Consequently, the correctness proof of min given in Bradley and Manna (2007, 2008) proves an algorithm that is different from ${\textsc {QX}}$. Moreover, the proof of min does not appear to be of great help to better understand the related ${\textsc {QX}}$ algorithm, as the reader needs to become familiar with the notation and concepts used in Bradley and Manna (2007, 2008) in the first place, and needs to map the pseudocode notation of Bradley and Manna (2007, 2008) to the largely different one adopted by Junker in the original ${\textsc {QX}}$-paper (Junker 2004).

**Fig. 1:**
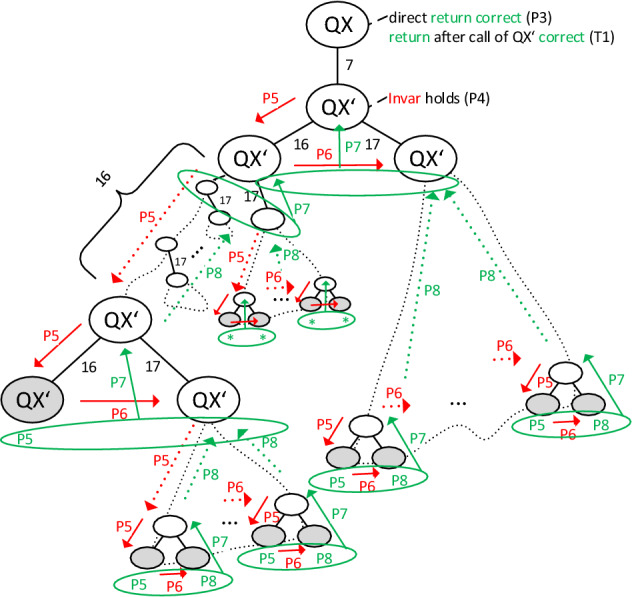
Call-recursion-tree produced by ${\textsc {QX}}$ (cf. Sect. 4). The *grayscale part of the figure* provides a schematic illustration of the procedure calls executed in a single run of ${\textsc {QX}}$ (where the recursion is entered, i.e., no trivial case applies). Each node (ellipse) represents one call of the procedure named within the ellipse. Edge labels (7,16,17) refer to the lines in Alg. 1 where the respective call is made. White ellipses (non-leaf nodes) are calls that issue further recursive calls (in lines 16 and 17), whereas gray ellipses (leaf nodes) are calls that directly return (i.e., in line 10 or 12). The *colored part of the figure* visualizes the meaning and consequences of the theorem (T1) and the various propositions (P*i*, for $i\in \{3,4,5,6,7,8\}$) that constitute the proof (cf. Sect. 5). Red arrows indicate proven propagations of the invariant property Invar (see Definition 4) between calls. Green arrows and labels indicate that respective calls return correct outputs. Start to read the colored illustrations from the top, just like ${\textsc {QX}}$ proceeds. That is, due to P3, direct returns yield correct outputs. If ${\textsc {QX}}'$ is called, Invar holds by P4. If Invar holds for some call, then it is always propagated downwards to the left subtree because of P5. At the first leaf node, a correct output is returned, also due to P5. If the output of a left subtree is correct, then Invar propagates to the right subtree (P6). If the output of both the left and the right subtree is correct, then the output of the root is correct (P7). If Invar holds at the root call of some (sub)tree, then this root call returns a correct output (P8). Note how these propositions guarantee that Invar, and thus correct outputs, can be derived for all nodes of the call-recursion-tree. Intuitively, red arrows propagate Invar downwards through the tree, which then ensures correct outcomes at the leafs, from where these correct outputs enable further propagation of Invar to the right, from where the inferred correct outputs are recursively propagated upwards until the root node is reached

**Fig. 2:**
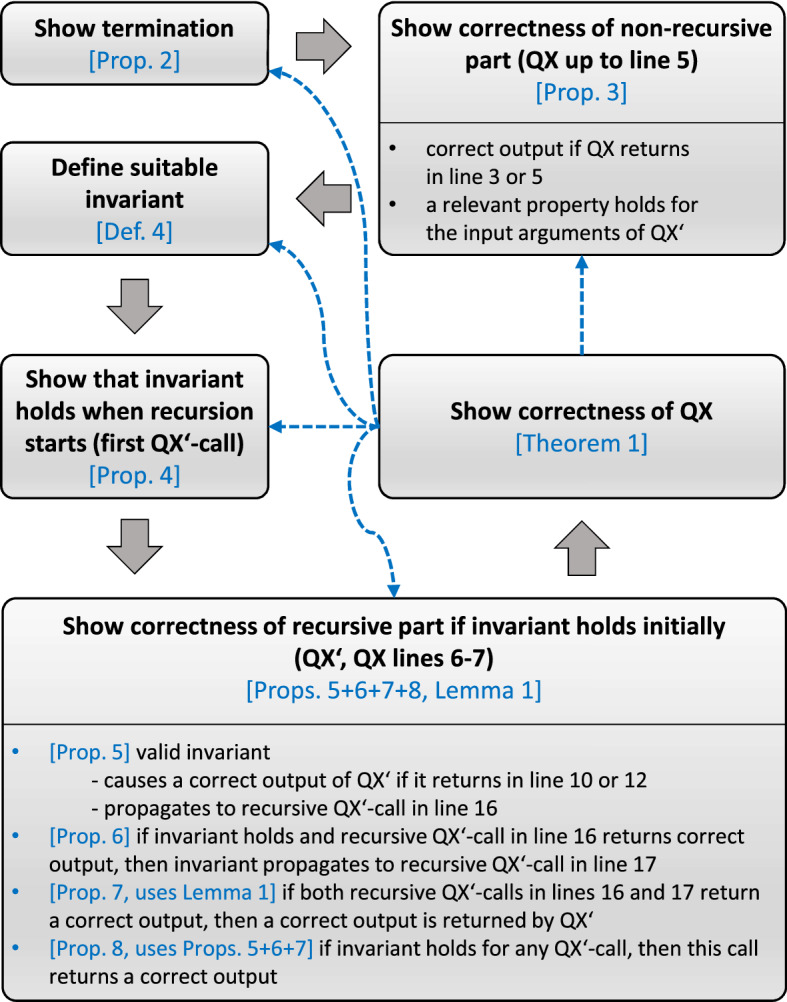
Summary of the main proof steps (boxes; bold black font) and associated propositions, lemma, and theorem (blue font). Gray arrows show the sequence of the proofs steps. Dashed blue arrows indicate dependencies, i.e., the step from where the arrow originates depends on the step to which the arrow points

In contrast, our proof does show the correctness of the ${\textsc {QX}}$ algorithm, and we present the algorithm in a very similar notation as used in Junker’s original paper (Junker 2004). Beyond that, the intention of our proof is to *prove and explain* ${\textsc {QX}}$ (Hanna 2000, 1990), rather than to solely prove it. To this end, e.g., we *(1)* segment our proof into small, intuitive, and easily digestible chunks, thus putting a special *focus on its clarity, elucidation, and didactic value*, *(2)* *provide visualizations* of the interrelations between and of the sequence and meaning of the individual proof steps by means of diagrams (cf. Figs. 1 and 2), *(3)* organize the proof in a way it is *illustrative and amenable to a mental “tracking”* in that it can be viewed as directly traversing the algorithm’s call-recursion-tree while verifying the correctness of all transitions in the tree (cf. Fig. 1), and *(4)* *explicate the proof template* adopted in our proof in order to promote the reader’s comprehension of the underlying general proof principle and to provide them with well-founded justifications for the individual proof steps (cf. Sect. 6).

## Basics

${\textsc {QX}}$ can be employed to find, for an input set *U*, a minimal^7^ subset $X \subseteq U$ that has a certain monotone property *p*. An example would be an (unsatisfiable) knowledge base (set of logical sentences) *U* for which we are interested in finding a minimal unsatisfiable subset (MUS) *X*.

### Definition 1

(Monotone Property) Let *U* be the universe (a set of elements) and $p:2^U \rightarrow \{0,1\}$ be a function where $p(X)=1$ iff property *p* holds for $X\subseteq U$. Then, *p* is a *monotone property* iff $p(\emptyset )=0$ and

$$\begin{aligned} \forall X', X'' \subseteq U:\;\, X' \subset X'' \implies p(X') \le p(X'') \end{aligned}$$

So, *p* is monotone iff, given that *p* holds for some set $X'$, it follows that *p* also holds for any superset $X''$ of $X'$. An equivalent definition is: If *p* does not hold for some set $X''$, *p* does not hold for any subset $X'$ of $X''$ either.

In practical applications it is often a requirement that *(a)* some elements of the universe must not occur in the sought minimal subset, or *(b)* the minimal subset of the universe should be found in the context of some reference set. Both cases (a) and (b) can be subsumed as searching for a minimal subset of the *analyzed set* ${\mathcal {A}}$ given some *background* ${\mathcal {B}}$. In case (a), ${\mathcal {B}}$ is defined as a subset of the universe *U* (e.g., in a fault localization task, those sentences of a knowledge base *U* that are assumed to be correct) and ${\mathcal {A}}$ is constituted by all other elements of the universe $U\setminus {\mathcal {B}}$ (those sentences in *U* that are possibly faulty); in case (b), ${\mathcal {B}}$ is some additional set of relevance to the universe (e.g., a knowledge base of general medical knowledge), whereas ${\mathcal {A}}$ is the universe itself (e.g., a knowledge base describing a medical sub-discipline). For example, the problem of finding a MUS wrt. ${\mathcal {A}}$ given background ${\mathcal {B}}$ would be to search for a minimal set *X* of elements in ${\mathcal {A}}$ such that $X \cup {\mathcal {B}}$ is unsatisfiable.

### Definition 2

(*p*-Problem-Instance) Let ${\mathcal {A}}$ (analyzed set) and ${\mathcal {B}}$ (background) be (related) finite sets of elements where ${\mathcal {A}}\cap {\mathcal {B}}= \emptyset$, and let *p* be a monotone predicate. Then we call the tuple $\left\langle {\mathcal {A}},{\mathcal {B}}\right\rangle$ a *p*-*problem-instance (p-PI)*.

### Definition 3

(Minimal *p*-Set (given some Background)) Let $\left\langle {\mathcal {A}},{\mathcal {B}}\right\rangle$ be a *p*-PI. Then, we call *X* a *p-set wrt.* $\left\langle {\mathcal {A}},{\mathcal {B}}\right\rangle$ iff $X\subseteq {\mathcal {A}}$ and $p(X \cup {\mathcal {B}})=1$. We call a *p*-set *X* wrt. $\left\langle {\mathcal {A}},{\mathcal {B}}\right\rangle$ *minimal* iff there is no *p*-set $X' \subset X$ wrt. $\left\langle {\mathcal {A}},{\mathcal {B}}\right\rangle$.

Immediate consequences of Definitions 1 and 3 are:

### Proposition 1

(Existence of a *p*-Set)

1. *A (minimal)* *p*-*set exists for* $\left\langle {\mathcal {A}},{\mathcal {B}}\right\rangle$ *iff* $p({\mathcal {A}}\cup {\mathcal {B}})=1$.
2. $\emptyset$ *is a—and the only—(minimal)* *p*-*set wrt.* $\left\langle {\mathcal {A}},{\mathcal {B}}\right\rangle$ *iff* $p({\mathcal {B}})=1$.^8^

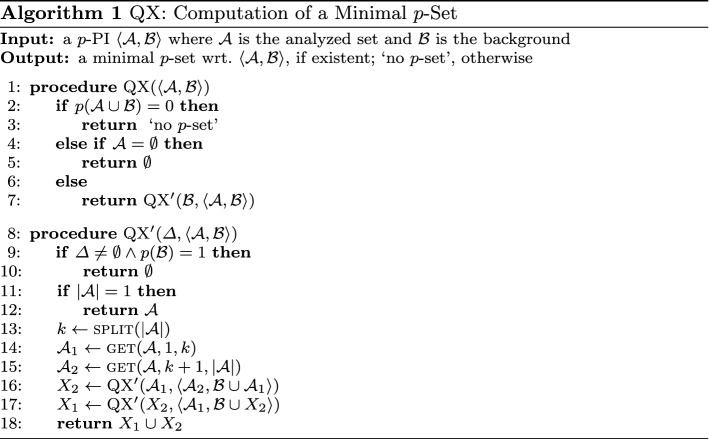


## Brief review and simple explanation of QX

The ${\textsc {QX}}$ algorithm is depicted by Alg. 1. It gets as input a *p*-PI $\left\langle {\mathcal {A}},{\mathcal {B}}\right\rangle$ and assumes a sound and complete oracle that answers queries of the form *p*(*X*) for arbitrary $X \subseteq {\mathcal {A}}\cup {\mathcal {B}}$.^9^ If existent, ${\textsc {QX}}$ returns a minimal *p*-set wrt. $\left\langle {\mathcal {A}},{\mathcal {B}}\right\rangle$; otherwise, ’no *p*-set’ is output. Note, in order to not overload the discussion, we focus in this work on QX’s property of computing a *minimal* *p*-set (instead of a preferred one, as in the original paper (Junker 2004)). That is, Alg. 1 does not include line 6 of the original algorithm. The minimality property of QX is key to solving the general MSMP problem which has numerous manifestations in a wide variety of research and application fields, as outlined in Sect. 1. In a nutshell, ${\textsc {QX}}$ works as follows:

*Trivial Cases:* Before line 7 is reached, the algorithm checks if trivial cases apply, i.e., if either no *p*-set exists (line 2; cf. Proposition 1.(1)) or a *p*-set does exist and the analyzed set ${\mathcal {A}}$ is empty (line 4), and returns according outputs. In case the execution reaches line 7, the recursive procedure ${\textsc {QX}}'$ is called. In the very first execution of ${\textsc {QX}}'$, the presence of two other trivial cases for the original input *p*-PI $\left\langle {\mathcal {A}},{\mathcal {B}}\right\rangle$ is checked in lines 9 and 11.^10^ (Line 9): If $p({\mathcal {B}})=1$, then the empty set, the only minimal *p*-set in this case (cf. Proposition 1.(2)), is directly returned and ${\textsc {QX}}$ terminates.^11^ Otherwise, we know the empty set is not a *p*-set, i.e., every (minimal) *p*-set is non-empty. (Line 11): If the analyzed set ${\mathcal {A}}$ is a singleton, then ${\mathcal {A}}$ is directly returned and ${\textsc {QX}}$ terminates.

*Recursion:* Subsequently, the recursion is started. The principle is to partition the analyzed set ${\mathcal {A}}= \{a_1,\dots ,a_{|{\mathcal {A}}|}\}$ into two *non-empty* (e.g., equal-sized) subsets ${\mathcal {A}}_1 = \{a_1,\dots ,a_k\}$ and ${\mathcal {A}}_2=\{a_{k+1},\dots ,a_{|{\mathcal {A}}|}\}$ (split and get functions; lines 13–15), and to analyze these subsets recursively (*divide-and-conquer*). In this vein, a binary call-recursion-tree is built (as sketched by the grayscale part of Fig. 1), including the *root* ${\textsc {QX}}'$-call made in line 7 and *two subtrees*, the left one rooted at the call of ${\textsc {QX}}'$ in line 16 which analyzes ${\mathcal {A}}_2$, and the right one rooted at the call of ${\textsc {QX}}'$ in line 17 which analyzes ${\mathcal {A}}_1$. Let the finally returned minimal *p*-set be denoted by *X*, and let us call all elements of *X* *relevant*, all others *irrelevant*. Then, the left subtree (finally) returns the subset of those elements ($X_2$) from ${\mathcal {A}}_2$ that belong to *X*, and the right subtree (finally) returns the subset of those elements ($X_1$) from ${\mathcal {A}}_1$ that belong to *X*.

- *Arguments of the recursive procedure* ${\textsc {QX}}'$*:* The arguments $\varDelta ,\left\langle {\mathcal {A}},{\mathcal {B}}\right\rangle$ passed to the procedure ${\textsc {QX}}'$ can be intuitively understood as follows. $\left\langle {\mathcal {A}},{\mathcal {B}}\right\rangle$ is the *p*-PI analyzed by the respective ${\textsc {QX}}'$-call. $\varDelta$ is (only^12^) relevant when ${\textsc {QX}}'$ was called in line 17 and essentially indicates whether some relevant element was found while analyzing ${\mathcal {A}}_2$ in the left subtree (${\textsc {QX}}'$-call in line 16). If so ($\varDelta = X_2 \ne \emptyset$), then $\varDelta$ “activates” the test ($p({\mathcal {B}})=1$?) in line 9 that checks if an exploration of the right subtree (${\mathcal {A}}_1$) is superfluous (or, in other words, if a full minimal *p*-set is already contained in ${\mathcal {A}}_2$). Otherwise ($\varDelta = X_2 = \emptyset$; no relevant element in ${\mathcal {A}}_2$), $\varDelta$ “deactivates” this check since a relevant element *must* be included in ${\mathcal {A}}_1$ (because at least one of ${\mathcal {A}}_1$ and ${\mathcal {A}}_2$ must include a relevant element), and therefore returning $\emptyset$ in line 10 as a result for ${\mathcal {A}}_1$ must be precluded.
- *Left subtree (recursive* ${\textsc {QX}}'$*-call in line 16):* The first question is: Are all elements of ${\mathcal {A}}_2$ irrelevant? Or, equivalently: Does ${\mathcal {B}}\cup {\mathcal {A}}_1$ already contain a minimal *p*-set, i.e., $p({\mathcal {B}}\cup {\mathcal {A}}_1)=1$? This is evaluated in line 9; note: $\varDelta = {\mathcal {A}}_1 \ne \emptyset$. If positive, $\emptyset$ is returned and the subtree is not further expanded. Otherwise, we know there is some relevant element in ${\mathcal {A}}_2$. Hence, the analysis of ${\mathcal {A}}_2$ is started. That is, in line 11, the singleton test is performed for ${\mathcal {A}}_2$. In the affirmative case, we have proven that the single element in ${\mathcal {A}}_2$ is relevant. The reason is that $p({\mathcal {B}}\cup {\mathcal {A}}_1) = 0$, as verified in line 9 just before, and that adding the single element in ${\mathcal {A}}_2$ makes the predicate true,^13^ i.e., $p({\mathcal {B}}\cup {\mathcal {A}}_1\cup {\mathcal {A}}_2) = p({\mathcal {B}}\cup {\mathcal {A}}) = 1$, as verified in line 2 at the very beginning. If ${\mathcal {A}}_2$ is a non-singleton, it is again partitioned and the subsets are analyzed recursively, which results in two new subtrees in the call-recursion-tree.
- *Right subtree (recursive* ${\textsc {QX}}'$*-call in line 17):* Here, we can distiguish between two possible cases, i.e., either the set $X_2$ returned by the left subtree is *(i)* empty or *(ii)* non-empty.
  Given (i), we know that ${\mathcal {A}}_1$ must include a relevant element. Reason: ${\mathcal {B}}\cup {\mathcal {A}}_1$ contains a minimal *p*-set (as verified in the left subtree before returning the empty set) and every *p*-set is non-empty (as verified in line 9 in the course of checking the *Trivial Cases*, see above). Hence, ${\mathcal {A}}_1$ is further analyzed in lines 11 et seqq. (which might lead to a direct return if ${\mathcal {A}}_1$ is a singleton and thus relevant, or to further recursive subtrees otherwise).
  For (ii), the question is: Given the subset $X_2$ of the *p*-set, are all elements of ${\mathcal {A}}_1$ irrelevant? Or, equivalently: Does ${\mathcal {B}}\cup X_2$ already contain a minimal *p*-set, i.e., $p({\mathcal {B}}\cup X_2)=1$? This is answered in line 9; note: $\varDelta = X_2 \ne \emptyset$ due to case (ii). In the affirmative case, the empty set is returned, i.e., no elements of ${\mathcal {A}}_1$ are relevant and the final *p*-set *X* found by ${\textsc {QX}}$ is equal to $X_2$. If the answer is negative, ${\mathcal {A}}_1$ does include some relevant element and is thus further analyzed in lines 11 et seqq. (which might lead to a direct return if ${\mathcal {A}}_1$ is a singleton and thus relevant, or to further recursive subtrees otherwise).

Finally, the union of the outcomes of left ($X_2$) and right ($X_1$) subtrees is a minimal *p*-set wrt. $\left\langle {\mathcal {A}},{\mathcal {B}}\right\rangle$ and returned in line 18.

### Example 1

We illustrate the functioning of ${\textsc {QX}}$ by means of a simple example.

*Input Problem and Parameter Setting:* Assume the analyzed set ${\mathcal {A}}= \{1,2,3,4,$ $5,6,7,8\}$, the initial background ${\mathcal {B}}= \emptyset$, and that there are two minimal *p*-sets wrt. $\left\langle {\mathcal {A}},{\mathcal {B}}\right\rangle$, $X=\{3,4,7\}$ and $Y=\{4,5,8\}$. Further, suppose that ${\textsc {QX}}$ pursues a splitting strategy where a set is always partitioned into equal-sized subsets in each iteration, i.e., $\textsc {split}(n)$ returns $\lceil \frac{n}{2}\rceil$ (note: this leads to the best worst-case complexity of ${\textsc {QX}}$, cf. Junker (2004)).

*Notation:* Below, we show the workings of ${\textsc {QX}}$ on this example by means of a tried and tested “flat” notation.^14^ In this notation, the single-underlined subset denotes the current input to the function *p* in line 9, the double-underlined elements are those that are already fixed elements of the returned minimal *p*-set, and the grayed out elements those that are definitely not in the returned minimal *p*-set. Finally, $\textcircled {1}$ signifies that the tested set (single-underlined along with double-underlined elements) is a *p*-set (function *p* in line 9 returns 1); $\textcircled {0}$ means it is no *p*-set (function *p* in line 9 returns 0).^15^

*How* ${\textsc {QX}}$ *Proceeds:* After verifying that there is a non-empty *p*-set wrt. $\left\langle {\mathcal {A}},{\mathcal {B}}\right\rangle$ and that $|{\mathcal {A}}|>1$ (i.e., after the checks in lines 2 and 4 are negative, ${\textsc {QX}}'$ is called in line 7, and the checks in the first execution of lines 9 and 11 are negative), ${\textsc {QX}}$ performs the following actions: 
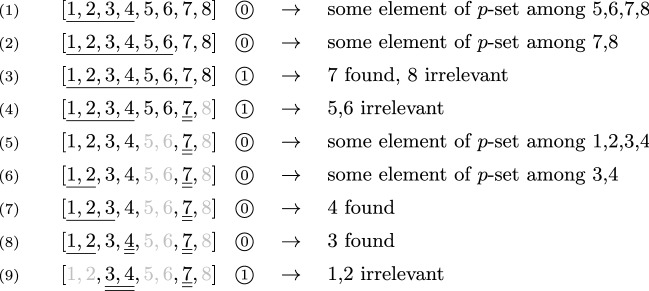


*Explanation:* After splitting ${\mathcal {A}}$ into two subsets of equal size, in step (1), ${\textsc {QX}}$ tests if there is a *p*-set in the left half $\{1,2,3,4\}$. Since negative, the right half $\{5,6,7,8\}$ is again split into equal-sized subsets, and the left one $\{5,6\}$ is added to the left half $\{1,2,3,4\}$ of the original set. Because this larger set $\{1,2,3,4,5,6\}$ still does not contain any *p*-set, the right subset $\{7,8\}$ is again split and the left part (7) added to the tested set, yielding $\{1,2,3,4,5,6,7\}$. Due to the positive predicate-test for this set, 7 is confirmed as an element of the found minimal *p*-set, and 8 is irrelevant. From now on, 7, as a fixed element of the *p*-set, takes part in all further executed predicate tests.

In step (4), the goal is to figure out whether the left half $\{5,6\}$ of $\{5,6,7,8\}$ also contains relevant elements. To this end, the left half $\{1,2,3,4\}$ of ${\mathcal {A}}$, along with 7, is tested, and positive. Therefore, a *p*-set is included in $\{1,2,3,4,7\}$ and $\{5,6\}$ is irrelevant. At this point, the output of the left subtree of the root, the one that analyzed $\{5,6,7,8\}$, is determined and fixed, i.e., is given by 7. The next task is to find the relevant elements in the right subtree, i.e., among $\{1,2,3,4\}$. As a consequence, in step (5), 7 alone is tested to check if all elements of $\{1,2,3,4\}$ are irrelevant. The result is negative, which is why the left half is split, and the left subset $\{1,2\}$ is tested along with 7, also negative. Thus, $\{3,4\}$ does include relevant elements. In step (7), ${\textsc {QX}}$ finds that the element 3 alone from the set $\{3,4\}$ does not suffice to produce a *p*-set, i.e., the test for $\{1,2,3,7\}$ is negative. This lets us conclude that 4 must be in the *p*-set. So, 4 is fixed. To check the relevance of 3, $\{1,2,4,7\}$ is tested, yielding a negative result, which proves that 3 is relevant. The final test in step (9) if $\{1,2\}$ includes relevant elements as well, is negative, and 1,2 marked irrelevant. The set $\{3,4,7\}$ is finally returned, which coincides with *X*, one of our minimal *p*-sets. $\square$

## Proof of QX

In this section, we give a formal proof^16^ of the termination and soundness of the ${\textsc {QX}}$ algorithm depicted by Alg. 1. By “soundness” we refer to the property that ${\textsc {QX}}$ outputs a minimal *p*-set wrt. the *p*-PI it gets as an input, if a *p*-set exists, and ’no *p*-set’ otherwise. While reading and thinking through the proof, the reader might consider it insightful to keep track of the meaning, implications, and interrelations of the various propositions in the proof by means of Fig. 1. Moreover, Fig. 2 summarizes the steps of the proof in a flowchart-like diagram.

### Proposition 2

(Termination) *Let* $\left\langle {\mathcal {A}},{\mathcal {B}}\right\rangle$ *be a* *p*-*PI. Then* ${\textsc {QX}}(\left\langle {\mathcal {A}},{\mathcal {B}}\right\rangle )$ *terminates*.^17^

### Proof

First, observe that ${\textsc {QX}}$ either reaches line 7 (where ${\textsc {QX}}'$ is called) or terminates before (in line 3 or line 5). Hence, ${\textsc {QX}}(\left\langle {\mathcal {A}},{\mathcal {B}}\right\rangle )$ always terminates iff ${\textsc {QX}}'({\mathcal {B}},\left\langle {\mathcal {A}},{\mathcal {B}}\right\rangle )$ always terminates. We next show that ${\textsc {QX}}'({\mathcal {B}},\left\langle {\mathcal {A}},{\mathcal {B}}\right\rangle )$ terminates for an arbitrary *p*-PI $\left\langle {\mathcal {A}},{\mathcal {B}}\right\rangle$.

${\textsc {QX}}'({\mathcal {B}},\left\langle {\mathcal {A}},{\mathcal {B}}\right\rangle )$ either terminates directly (in that it returns in line 10 or line 12) or calls itself recursively in lines 16 and 17. However, for each recursive call ${\textsc {QX}}'(\varDelta ',\left\langle {\mathcal {A}}',{\mathcal {B}}'\right\rangle )$ within ${\textsc {QX}}'({\mathcal {B}},\left\langle {\mathcal {A}},{\mathcal {B}}\right\rangle )$ it holds that $\emptyset \subset {\mathcal {A}}' \subset {\mathcal {A}}$ as ${\mathcal {A}}' \in \left\{ {\mathcal {A}}_1,{\mathcal {A}}_2\right\}$ (see lines 14 and 15) and $\emptyset \subset {\mathcal {A}}_1, {\mathcal {A}}_2 \subset {\mathcal {A}}$ due to the definition of the split and get functions.

Now, assume an infinite sequence of nested recursive calls of ${\textsc {QX}}'$. Since ${\mathcal {A}}$ is finite (Definition 2), this means that there must be a call ${\textsc {QX}}'({\bar{\varDelta }},\left\langle {\bar{{\mathcal {A}}}},{\bar{{\mathcal {B}}}}\right\rangle )$ in this sequence where $|{\bar{{\mathcal {A}}}}|=1$ and lines 16 and 17 (next nested recursive call in the infinite sequence) are reached. This is a contradiction to the fact that the test in line 11 enforces a return in line 12 given that $|{\bar{{\mathcal {A}}}}|=1$. Consequently, every sequence of nested recursive calls during the execution of ${\textsc {QX}}'({\mathcal {B}},\left\langle {\mathcal {A}},{\mathcal {B}}\right\rangle )$ is finite (i.e., the depth of the call tree is finite).

Finally, there can only be a finite number of such nested recursive call sequences because no more than two recursive calls are made in any execution of ${\textsc {QX}}'$ (i.e., the branching factor of the call tree is 2). This completes the proof. $\square$

The following proposition witnesses that ${\textsc {QX}}$ is sound in case the sub-procedure ${\textsc {QX}}'$ is never called.

### Proposition 3

(Correctness of ${\textsc {QX}}$ When Trivial Cases Apply)

1. ${\textsc {QX}}(\left\langle {\mathcal {A}},{\mathcal {B}}\right\rangle )$ *returns ’no* *p*-set’ *in line* 3 *iff there is no* *p*-*set wrt.* $\left\langle {\mathcal {A}},{\mathcal {B}}\right\rangle$.
2. *If* ${\textsc {QX}}(\left\langle {\mathcal {A}},{\mathcal {B}}\right\rangle )$ *returns* $\emptyset$ *in line* 5, $\emptyset$ *is a minimal* *p*-*set wrt.* $\left\langle {\mathcal {A}},{\mathcal {B}}\right\rangle$.
3. *If the execution of* ${\textsc {QX}}(\left\langle {\mathcal {A}},{\mathcal {B}}\right\rangle )$ *reaches line* 7, $p({\mathcal {A}}\cup {\mathcal {B}})=1$ *holds.*

### Proof

We prove all statements (1)–(3) in turn.

*Proof of (1):* The fact follows directly from Proposition 1.(1) and the test performed in line 2.

*Proof of (2):* Because line 5 is reached, $p({\mathcal {A}}\cup {\mathcal {B}})=1$ (as otherwise a return would have taken place at line 3) and ${\mathcal {A}}= \emptyset$ (due to line 4) must hold. Since $p({\mathcal {A}}\cup {\mathcal {B}})=1$ implies the existence of a *p*-set wrt. $\left\langle {\mathcal {A}},{\mathcal {B}}\right\rangle$ by Proposition 1.(1), and since any *p*-set wrt. $\left\langle {\mathcal {A}},{\mathcal {B}}\right\rangle$ must be a subset of ${\mathcal {A}}$ by Definition 3, $\emptyset$ is the only (and therefore trivially a minimal) *p*-set wrt. $\left\langle {\mathcal {A}},{\mathcal {B}}\right\rangle$.

*Proof of (3):* This statement follows directly from the test in line 2 and the fact that line 7 is reached. $\square$

We now characterize an invariant which applies to every call of ${\textsc {QX}}'$ throughout the execution of ${\textsc {QX}}$.

### Definition 4

(Invariant Property of ${\textsc {QX}}'$) Let ${\textsc {QX}}'(\varDelta ,\left\langle {\mathcal {A}},{\mathcal {B}}\right\rangle )$ be a call of ${\textsc {QX}}'$. Then we say that $\mathsf {Invar}(\varDelta ,{\mathcal {A}},{\mathcal {B}})$ holds for this call iff

$$\begin{aligned} (\varDelta \ne \emptyset \vee p({\mathcal {B}})=0) \;\,\wedge \;\, p({\mathcal {A}}\cup {\mathcal {B}}) = 1 \end{aligned}$$

The next proposition shows that this invariant holds for the first call of ${\textsc {QX}}'$ in Alg. 1.

### Proposition 4

(Invariant Holds For First Call of ${\textsc {QX}}'$) $\mathsf {Invar}({\bar{\varDelta }},{\bar{{\mathcal {A}}}},{\bar{{\mathcal {B}}}})$ *holds for* ${\textsc {QX}}'({\bar{\varDelta }},\left\langle {\bar{{\mathcal {A}}}},{\bar{{\mathcal {B}}}}\right\rangle )$ *given that* ${\textsc {QX}}'({\bar{\varDelta }},\left\langle {\bar{{\mathcal {A}}}},{\bar{{\mathcal {B}}}}\right\rangle )$ *was called in line* 7.

### Proof

Since ${\textsc {QX}}'({\bar{\varDelta }},\left\langle {\bar{{\mathcal {A}}}},{\bar{{\mathcal {B}}}}\right\rangle )$ was called in line 7, we have ${\bar{\varDelta }}= {\mathcal {B}}$, ${\bar{{\mathcal {A}}}}= {\mathcal {A}}$ and ${\bar{{\mathcal {B}}}}= {\mathcal {B}}$. Since $p({\mathcal {A}}\cup {\mathcal {B}})=1$ holds in line 7 on account of Proposition 3.(3), we have that $p({\bar{{\mathcal {A}}}}\cup {\bar{{\mathcal {B}}}})=1$. To show that $({\bar{\varDelta }}\ne \emptyset \vee p({\bar{{\mathcal {B}}}})=0)$, we distinguish the cases ${\mathcal {B}}= \emptyset$ and ${\mathcal {B}}\ne \emptyset$. Let first ${\mathcal {B}}= \emptyset$. Due to Definition 1, we have that $p({\bar{{\mathcal {B}}}})=p({\mathcal {B}})=p(\emptyset )=0$. Second, assume ${\mathcal {B}}\ne \emptyset$. Since ${\bar{\varDelta }}= {\mathcal {B}}$, we directly obtain that ${\bar{\varDelta }}\ne \emptyset$. $\square$

Given the invariant of Definition 4 holds for some call of ${\textsc {QX}}'$, we next demonstrate that the output returned by ${\textsc {QX}}'$ is sound (i.e., a minimal *p*-set) when it returns in line 10 or 12 (i.e., if this call of ${\textsc {QX}}'$ represents a leaf node in the call-recursion-tree). Moreover, we show that the invariant is “propagated” to the recursive call of ${\textsc {QX}}'$ in line 16 (i.e., this invariant remains valid as long as the algorithm keeps going downwards in the call-recursion-tree).

### Proposition 5

(Invariant Causes Sound Outputs and Propagates Downwards) *If* $\mathsf {Invar}(\varDelta ,{\mathcal {A}},{\mathcal {B}})$ *holds for* ${\textsc {QX}}'(\varDelta ,\left\langle {\mathcal {A}},{\mathcal {B}}\right\rangle )$, then:

1. ${\textsc {QX}}'(\varDelta ,\left\langle {\mathcal {A}},{\mathcal {B}}\right\rangle )$ *returns* $\emptyset$ *in line* 10 *iff* $\emptyset$ *is a (minimal)* *p*-*set wrt.* $\left\langle {\mathcal {A}},{\mathcal {B}}\right\rangle$.
2. *If the execution of* ${\textsc {QX}}'(\varDelta ,\left\langle {\mathcal {A}},{\mathcal {B}}\right\rangle )$ *reaches line* 11, *then* $p({\mathcal {B}})=0$ *holds.*
3. *If* ${\textsc {QX}}'(\varDelta ,\left\langle {\mathcal {A}},{\mathcal {B}}\right\rangle )$ *returns* ${\mathcal {A}}$ *in line* 12, *then* ${\mathcal {A}}$ *is a minimal* *p*-*set wrt.* $\left\langle {\mathcal {A}},{\mathcal {B}}\right\rangle$.
4. *If the execution of* ${\textsc {QX}}'(\varDelta ,\left\langle {\mathcal {A}},{\mathcal {B}}\right\rangle )$ *reaches line* 16, *where* ${\textsc {QX}}'({\bar{\varDelta }},\left\langle {\bar{{\mathcal {A}}}},{\bar{{\mathcal {B}}}}\right\rangle )$ *is called, then* $\mathsf {Invar}({\bar{\varDelta }},{\bar{{\mathcal {A}}}},{\bar{{\mathcal {B}}}})$.

### Proof

We prove all statements (1)–(4) in turn.

*Proof of (1):* “$\Rightarrow$”: We assume that ${\textsc {QX}}'(\varDelta ,\left\langle {\mathcal {A}},{\mathcal {B}}\right\rangle )$ returns in line 10. By the test performed in line 9, this can only be the case if $p({\mathcal {B}})=1$. By Proposition 1.(2), this implies that $\emptyset$ is a (minimal) *p*-set wrt. $\left\langle {\mathcal {A}},{\mathcal {B}}\right\rangle$.

“$\Leftarrow$”: We assume that $\emptyset$ is a (minimal) *p*-set wrt. $\left\langle {\mathcal {A}},{\mathcal {B}}\right\rangle$. To show that a return takes place in line 10, we have to prove that the condition tested in line 9 is true. First, we observe that $p({\mathcal {B}})=1$ must hold due to Proposition 1.(2). Since $\mathsf {Invar}(\varDelta ,{\mathcal {A}},{\mathcal {B}})$ holds (see Definition 4), we can infer from $p({\mathcal {B}})=1$ that $\varDelta \ne \emptyset$. Hence, the condition in line 9 is satisfied.

*Proof of (2):* Proposition 5.(1) shows that line 11 is reached iff $\emptyset$ is not a *p*-set wrt. $\left\langle {\mathcal {A}},{\mathcal {B}}\right\rangle$ which is the case iff $p({\mathcal {B}})=0$ due to Proposition 1.(2).

*Proof of (3):* A return in line 12 can only occur if the test in line 11 is positive, i.e., if line 11 is reached and $|{\mathcal {A}}|=1$. Moreover, since $\mathsf {Invar}(\varDelta ,{\mathcal {A}},{\mathcal {B}})$ holds, it follows that $p({\mathcal {A}}\cup {\mathcal {B}})=1$.

First, $p({\mathcal {A}}\cup {\mathcal {B}})=1$ is equivalent to the existence of a *p*-set wrt. $\left\langle {\mathcal {A}},{\mathcal {B}}\right\rangle$. Second, by Definition 3, a *p*-set wrt. $\left\langle {\mathcal {A}},{\mathcal {B}}\right\rangle$ is a subset of ${\mathcal {A}}$. Third, $|{\mathcal {A}}|=1$ means that $\emptyset$ and ${\mathcal {A}}$ are all possible subsets of ${\mathcal {A}}$. Fourth, since line 11 is reached, we have that $p({\mathcal {B}})=0$ by statement (2) of this Proposition, which implies that $\emptyset$ is not a *p*-set wrt. $\left\langle {\mathcal {A}},{\mathcal {B}}\right\rangle$ according to Proposition 1.(2). Consequently, ${\mathcal {A}}$ must be a minimal *p*-set wrt. $\left\langle {\mathcal {A}},{\mathcal {B}}\right\rangle$.

*Proof of (4):* Consider the call ${\textsc {QX}}'({\bar{\varDelta }},\left\langle {\bar{{\mathcal {A}}}},{\bar{{\mathcal {B}}}}\right\rangle )$ at line 16. Due to the definition of the split and get functions ($1\le k \le |{\mathcal {A}}|-1$, ${\mathcal {A}}_1$ includes the first *k*, ${\mathcal {A}}_2$ the last $|{\mathcal {A}}|-k$ elements of ${\mathcal {A}}$) and the fact that ${\bar{\varDelta }}= {\mathcal {A}}_1$, the property ${\bar{\varDelta }}\ne \emptyset$ must hold. Moreover, ${\bar{{\mathcal {A}}}}\cup {\bar{{\mathcal {B}}}}= {\mathcal {A}}_2\cup {\mathcal {B}}\cup {\mathcal {A}}_1 = {\mathcal {A}}\cup {\mathcal {B}}$. Due to $\mathsf {Invar}(\varDelta ,{\mathcal {A}},{\mathcal {B}})$, however, we know that $p({\mathcal {A}}\cup {\mathcal {B}})=1$. Therefore, $p({\bar{{\mathcal {A}}}}\cup {\bar{{\mathcal {B}}}})=1$ must be true. According to Definition 4, it follows that $\mathsf {Invar}({\bar{\varDelta }},{\bar{{\mathcal {A}}}},{\bar{{\mathcal {B}}}})$ holds. $\square$

Note, immediately before line 17 is first reached during the execution of ${\textsc {QX}}$, it must be the case that, for the first time, a recursive call ${\textsc {QX}}'(\varDelta ,\left\langle {\mathcal {A}},{\mathcal {B}}\right\rangle )$ made in line 16 returns (i.e., we reach a leaf node in the call-recursion-tree for the first time and the first “backtracking” takes place). By Proposition 5.(1)+(3), the output of this call ${\textsc {QX}}'(\varDelta ,\left\langle {\mathcal {A}},{\mathcal {B}}\right\rangle )$, namely $X_2$ in line 16, is a minimal *p*-set wrt. $\left\langle {\mathcal {A}},{\mathcal {B}}\right\rangle$. We now prove that the invariant property given in Definition 4 in this case “propagates” to the first-ever call of ${\textsc {QX}}'$ in line 17.

### Proposition 6

(If Output of Left Subtree is Sound, Invariant Propagates to Right Subtree) *Let* $\mathsf {Invar}(\varDelta ,{\mathcal {A}},{\mathcal {B}})$ *be true for some call* ${\textsc {QX}}'(\varDelta ,\left\langle {\mathcal {A}},{\mathcal {B}}\right\rangle )$ *and let the recursive call* ${\textsc {QX}}'({\dot{\varDelta }},\langle {\dot{{\mathcal {A}}}},{\dot{{\mathcal {B}}}}\rangle )$ *in line* 16 *during the execution of* ${\textsc {QX}}'(\varDelta ,\left\langle {\mathcal {A}},{\mathcal {B}}\right\rangle )$ *return a minimal* *p*-*set wrt.* $\langle {\dot{{\mathcal {A}}}},{\dot{{\mathcal {B}}}}\rangle$. *Then* $\mathsf {Invar}(\ddot{\varDelta },\ddot{{\mathcal {A}}},\ddot{{\mathcal {B}}})$ *holds for the recursive call* ${\textsc {QX}}'(\ddot{\varDelta },\langle \ddot{{\mathcal {A}}},\ddot{{\mathcal {B}}}\rangle )$ *in line 17 during the execution of* ${\textsc {QX}}'(\varDelta ,\left\langle {\mathcal {A}},{\mathcal {B}}\right\rangle )$.

### Proof

As per Definition 4, we have to show that $(\ddot{\varDelta }\ne \emptyset \vee p(\ddot{{\mathcal {B}}})=0) \wedge p(\ddot{{\mathcal {A}}}\cup \ddot{{\mathcal {B}}}) = 1$.

We first prove $p(\ddot{{\mathcal {A}}}\cup \ddot{{\mathcal {B}}}) = 1$. Since $X_2$, the set returned by ${\textsc {QX}}'({\dot{\varDelta }},\langle {\dot{{\mathcal {A}}}},{\dot{{\mathcal {B}}}}\rangle ) = {\textsc {QX}}'({\mathcal {A}}_1,\langle {\mathcal {A}}_2,{\mathcal {B}}\cup {\mathcal {A}}_1\rangle )$ in line 16, is a minimal *p*-set wrt. $\langle {\dot{{\mathcal {A}}}},{\dot{{\mathcal {B}}}}\rangle = \langle {\mathcal {A}}_2,{\mathcal {B}}\cup {\mathcal {A}}_1\rangle$, we infer by Definition 3 that $p(X_2 \cup {\mathcal {B}}\cup {\mathcal {A}}_1) = 1$. However, it holds that ${\textsc {QX}}'(\ddot{\varDelta },\langle \ddot{{\mathcal {A}}},\ddot{{\mathcal {B}}}\rangle )={\textsc {QX}}'(X_2,\left\langle {\mathcal {A}}_1,{\mathcal {B}}\cup X_2\right\rangle )$. Therefore, $p(\ddot{{\mathcal {A}}}\cup \ddot{{\mathcal {B}}}) = p([{\mathcal {A}}_1]\cup [{\mathcal {B}}\cup X_2]) = 1$.

It remains to be shown that $(\ddot{\varDelta }\ne \emptyset \vee p(\ddot{{\mathcal {B}}})=0)$ holds, which is equivalent to $(X_2 \ne \emptyset \vee p({\mathcal {B}}\cup X_2)=0)$. If $X_2 \ne \emptyset$, we are done. So, let us assume that $X_2 = \emptyset$. In this case, however, we have $p({\mathcal {B}}\cup X_2)=p({\mathcal {B}})$. As $\mathsf {Invar}(\varDelta ,\left\langle {\mathcal {A}},{\mathcal {B}}\right\rangle )$ holds and line 17 is reached during the execution of ${\textsc {QX}}'(\varDelta ,\left\langle {\mathcal {A}},{\mathcal {B}}\right\rangle )$, we know by Proposition 5.(2) that $p({\mathcal {B}})=0$. Hence, $p({\mathcal {B}}\cup X_2) = p({\mathcal {B}}) = 0$.

Overall, we have demonstrated that $\mathsf {Invar}(\ddot{\varDelta },\langle \ddot{{\mathcal {A}}},\ddot{{\mathcal {B}}}\rangle )$ holds. $\square$

At this point, we know that the invariant property of Definition 4 remains valid up to and including the first recursive call of ${\textsc {QX}}'$ in line 17 (i.e., until immediately after the first leaf in the call-recursion-tree is encountered, a single-step “backtrack” is made, and the first branching to the right is executed). From then on, as long as only “downward” calls of ${\textsc {QX}}'$ in line 16, possibly interleaved with single calls of ${\textsc {QX}}'$ in line 17, are performed, the validity of the invariant is preserved.

Due to the fact that ${\textsc {QX}}$ terminates (Proposition 2), the call-recursion-tree must be finite. Hence, the situation must occur, where ${\textsc {QX}}'$ called in line 16 directly returns (i.e., in line 10 or 12) and the immediately subsequent call of ${\textsc {QX}}'$ in line 17 directly returns (i.e., in line 10 or 12) as well (i.e., we face the situation where both the left and the right branch at one node in the call-recursion-tree consist only of a single leaf node). As the invariant holds in this right branch, the said call of ${\textsc {QX}}'$ in line 17 must indeed return a minimal *p*-set wrt. its *p*-PI given as an argument, due to Proposition 5.(1)+(3).

The next proposition evidences—as a special case—that the combination (set-union) of the two outputs $X_2$ (left leaf node) and $X_1$ (right leaf node) returned in line 18 in fact constitutes a minimal *p*-set for the *p*-PI given as an input argument to the call of ${\textsc {QX}}'$ which executes line 18. More generally, the proposition testifies that, given the calls in line 16 and line 17 each return a minimal *p*-set wrt. their given *p*-PIs—whether or not these calls directly return—the combination of these *p*-sets is again a minimal *p*-set for the respective *p*-PI at the call that executed lines 16 and 17.

### Proposition 7

(If Output of Both Left and Right Subtree is Sound, then a Sound Result is Returned (Propagated Upwards)) *Let the recursive call* ${\textsc {QX}}'({\dot{\varDelta }},\langle {\dot{{\mathcal {A}}}},{\dot{{\mathcal {B}}}}\rangle )$ *in line 16 during the execution of* ${\textsc {QX}}'({\bar{\varDelta }},\left\langle {\bar{{\mathcal {A}}}},{\bar{{\mathcal {B}}}}\right\rangle )$ *return a minimal* *p*-*set wrt.* $\langle {\dot{{\mathcal {A}}}},{\dot{{\mathcal {B}}}}\rangle$, *and let the recursive call* ${\textsc {QX}}'(\ddot{\varDelta },\langle \ddot{{\mathcal {A}}},\ddot{{\mathcal {B}}}\rangle )$ *in line 17 during the execution of* ${\textsc {QX}}'({\bar{\varDelta }},\left\langle {\bar{{\mathcal {A}}}},{\bar{{\mathcal {B}}}}\right\rangle )$ *return a minimal* *p*-set wrt. $\langle \ddot{{\mathcal {A}}},\ddot{{\mathcal {B}}}\rangle$. *Then* ${\textsc {QX}}'({\bar{\varDelta }},\left\langle {\bar{{\mathcal {A}}}},{\bar{{\mathcal {B}}}}\right\rangle )$ *returns a minimal* *p*-*set wrt.* $\left\langle {\bar{{\mathcal {A}}}},{\bar{{\mathcal {B}}}}\right\rangle$.

### Proof

The statement is a direct consequence of Lemma 1 below. $\square$

### Lemma 1

*Let* ${\mathcal {A}}_1, {\mathcal {A}}_2$ *be a partition of* ${\mathcal {A}}$. *If* *(a)* $X_2$ *is a minimal* *p*-*set wrt.* $\left\langle {\mathcal {A}}_2, {\mathcal {B}}\cup {\mathcal {A}}_1\right\rangle$ *and* *(b)* $X_1$ *is a minimal* *p*-*set wrt.* $\left\langle {\mathcal {A}}_1, {\mathcal {B}}\cup X_2\right\rangle$, *then* $X_1 \cup X_2$ *is a minimal* *p*-*set wrt.* $\left\langle {\mathcal {A}}, {\mathcal {B}}\right\rangle$.^18^

### Proof

We first show that $X_1 \cup X_2$ is a *p*-set, and then we show its minimality.

*p*-*Set Property:* First, by Definition 3, $X_1 \subseteq {\mathcal {A}}_1$ due to (a), and $X_2 \subseteq {\mathcal {A}}_2$ due to (b), which is why $X_1 \cup X_2 \subseteq {\mathcal {A}}_1 \cup {\mathcal {A}}_2 = {\mathcal {A}}$. From the fact that $X_1$ is a minimal *p*-set wrt. $\left\langle {\mathcal {A}}_1, {\mathcal {B}}\cup X_2\right\rangle$, along with Definition 3, we get $p(X_1 \cup [{\mathcal {B}}\cup X_2]) = 1 = p([X_1 \cup X_2] \cup {\mathcal {B}})$. Hence, $X_1 \cup X_2$ is a *p*-set wrt. $\left\langle {\mathcal {A}},{\mathcal {B}}\right\rangle$ due to Definition 3.

*Minimality:* To show that $X_1 \cup X_2$ is a *minimal* *p*-set wrt. $\left\langle {\mathcal {A}},{\mathcal {B}}\right\rangle$, assume that $X \subset X_1 \cup X_2$ is a *p*-set wrt. $\left\langle {\mathcal {A}},{\mathcal {B}}\right\rangle$. The set *X* can be represented as $X=X'_1 \cup X'_2$ where (1) $X'_1 := X \cap X_1 \subseteq X_1$ and (2) $X'_2 := X \cap X_2 \subseteq X_2$. In addition, the $\subseteq$-relation in (1) or (2) must be a $\subset$-relation, i.e., $X'_1 = X_1$ and $X'_2 = X_2$ cannot both hold.

Let us first assume that $\subset$ holds in (1). Then, $X= X'_1 \cup X'_2$ where $X'_1 \subset X_1$ and $X'_2 \subseteq X_2$. Since *X* is a *p*-set wrt. $\left\langle {\mathcal {A}},{\mathcal {B}}\right\rangle$, we have $p(X\cup {\mathcal {B}}) = p([X'_1 \cup X'_2] \cup {\mathcal {B}}) = p(X'_1 \cup [{\mathcal {B}}\cup X'_2])=1$. By monotonicity of *p*, it follows that $p(X'_1 \cup [{\mathcal {B}}\cup X_2])=1$. Because of $X'_1 \subset X_1 \subseteq {\mathcal {A}}_1$, we have that $X'_1$ is a *p*-set wrt. $\left\langle {\mathcal {A}}_1,{\mathcal {B}}\cup X_2\right\rangle$, which is a contradiction to the premise (b).

Second, assume that $\subset$ holds in (2). Then, $X= X'_1 \cup X'_2$ where $X'_1 \subseteq X_1$ and $X'_2 \subset X_2$. Since *X* is a *p*-set wrt. $\left\langle {\mathcal {A}},{\mathcal {B}}\right\rangle$, we have $p(X\cup {\mathcal {B}}) = p([X'_1 \cup X'_2] \cup {\mathcal {B}}) = p(X'_2 \cup [{\mathcal {B}}\cup X'_1])=1$. By monotonicity of *p*, and since $X'_1 \subseteq X_1 \subseteq {\mathcal {A}}_1$, it follows that $p(X'_2 \cup [{\mathcal {B}}\cup {\mathcal {A}}_1])=1$. As $X'_2 \subset X_2 \subseteq {\mathcal {A}}_2$, we obtain that $X'_2$ is a *p*-set wrt. $\left\langle {\mathcal {A}}_2,{\mathcal {B}}\cup {\mathcal {A}}_1\right\rangle$, which is a contradiction to premise (a). $\square$

### Proposition 8

(If Invariant Holds for Tree, Then a Minimal *p*-Set is Returned By Tree) *If* $\mathsf {Invar}({\bar{\varDelta }},\left\langle {\bar{{\mathcal {A}}}},{\bar{{\mathcal {B}}}}\right\rangle )$ *holds for* ${\textsc {QX}}'({\bar{\varDelta }},\left\langle {\bar{{\mathcal {A}}}},{\bar{{\mathcal {B}}}}\right\rangle )$, *then it returns a minimal* *p*-*set wrt.* $\left\langle {\bar{{\mathcal {A}}}},{\bar{{\mathcal {B}}}}\right\rangle$.

### Proof

We prove this proposition by induction on *d* where *d* is the maximal number of *recursive*^19^ calls of ${\textsc {QX}}'$ on the call stack throughout the execution of ${\textsc {QX}}'({\bar{\varDelta }},\left\langle {\bar{{\mathcal {A}}}},{\bar{{\mathcal {B}}}}\right\rangle )$.

*Induction Base:* Let $d=0$. That is, no recursive calls are executed, or, equivalently, ${\textsc {QX}}'({\bar{\varDelta }},\left\langle {\bar{{\mathcal {A}}}},{\bar{{\mathcal {B}}}}\right\rangle )$ returns in line 10 or 12. Since $\mathsf {Invar}({\bar{\varDelta }},\left\langle {\bar{{\mathcal {A}}}},{\bar{{\mathcal {B}}}}\right\rangle )$ is true, a minimal *p*-set wrt. $\left\langle {\bar{{\mathcal {A}}}},{\bar{{\mathcal {B}}}}\right\rangle$ is returned, which follows from Proposition 5.(1)+(3).

*Induction Assumption:* Let the statement of the proposition be true for $d=k$. We will now show that, in this case, the statement holds for $d = k+1$ as well.

*Induction Step:* Assume that (at most) $k+1$ recursive calls are ever on the call stack while ${\textsc {QX}}'({\bar{\varDelta }},\left\langle {\bar{{\mathcal {A}}}},{\bar{{\mathcal {B}}}}\right\rangle )$ executes. Since $\mathsf {Invar}({\bar{\varDelta }},\left\langle {\bar{{\mathcal {A}}}},{\bar{{\mathcal {B}}}}\right\rangle )$ holds, Proposition 5.(4) lets us conclude that $\mathsf {Invar}({\dot{\varDelta }},\langle {\dot{{\mathcal {A}}}},{\dot{{\mathcal {B}}}}\rangle )$ holds for the first recursive call ${\textsc {QX}}'({\dot{\varDelta }},\langle {\dot{{\mathcal {A}}}},{\dot{{\mathcal {B}}}}\rangle )$ issued in line 16 of ${\textsc {QX}}'({\bar{\varDelta }},\left\langle {\bar{{\mathcal {A}}}},{\bar{{\mathcal {B}}}}\right\rangle )$. Now, we have that, for ${\textsc {QX}}'({\dot{\varDelta }},\langle {\dot{{\mathcal {A}}}},{\dot{{\mathcal {B}}}}\rangle )$, the maximal number of recursive calls ever on the call stack while it executes, is (at most) *k*. Therefore, by the *Induction Assumption*, ${\textsc {QX}}'({\dot{\varDelta }},\langle {\dot{{\mathcal {A}}}},{\dot{{\mathcal {B}}}}\rangle )$ returns a minimal *p*-set wrt. $\langle {\dot{{\mathcal {A}}}},{\dot{{\mathcal {B}}}}\rangle$.

Because $\mathsf {Invar}({\bar{\varDelta }},\left\langle {\bar{{\mathcal {A}}}},{\bar{{\mathcal {B}}}}\right\rangle )$ holds and ${\textsc {QX}}'({\dot{\varDelta }},\langle {\dot{{\mathcal {A}}}},{\dot{{\mathcal {B}}}}\rangle )$ called in line 16 during the execution of ${\textsc {QX}}'({\bar{\varDelta }},\left\langle {\bar{{\mathcal {A}}}},{\bar{{\mathcal {B}}}}\right\rangle )$ returns a minimal *p*-set wrt. $\langle {\dot{{\mathcal {A}}}},{\dot{{\mathcal {B}}}}\rangle$, we deduce by means of Proposition 6 that $\mathsf {Invar}(\ddot{\varDelta },\langle \ddot{{\mathcal {A}}},\ddot{{\mathcal {B}}}\rangle )$ holds for the call ${\textsc {QX}}'(\ddot{\varDelta },\langle \ddot{{\mathcal {A}}},\ddot{{\mathcal {B}}}\rangle )$ made in line 17 during the execution of ${\textsc {QX}}'({\bar{\varDelta }},\left\langle {\bar{{\mathcal {A}}}},{\bar{{\mathcal {B}}}}\right\rangle )$. Again, it must be true that the maximal number of recursive calls ever on the call stack while ${\textsc {QX}}'(\ddot{\varDelta },\langle \ddot{{\mathcal {A}}},\ddot{{\mathcal {B}}}\rangle )$ executes is (at most) *k*. Consequently, ${\textsc {QX}}'(\ddot{\varDelta },\langle \ddot{{\mathcal {A}}},\ddot{{\mathcal {B}}}\rangle )$ returns a minimal *p*-set wrt. $\langle \ddot{{\mathcal {A}}},\ddot{{\mathcal {B}}}\rangle$ due to the *Induction Assumption*.

As both recursive calls made throughout the execution of ${\textsc {QX}}'({\bar{\varDelta }},\left\langle {\bar{{\mathcal {A}}}},{\bar{{\mathcal {B}}}}\right\rangle )$ return a minimal *p*-set wrt. their given *p*-PIs $\langle {\dot{{\mathcal {A}}}},{\dot{{\mathcal {B}}}}\rangle$ and $\langle \ddot{{\mathcal {A}}},\ddot{{\mathcal {B}}}\rangle$, respectively, we conclude by Proposition 7 that ${\textsc {QX}}'({\bar{\varDelta }},\left\langle {\bar{{\mathcal {A}}}},{\bar{{\mathcal {B}}}}\right\rangle )$ returns a minimal *p*-set wrt. $\left\langle {\bar{{\mathcal {A}}}},{\bar{{\mathcal {B}}}}\right\rangle$.

This completes the inductive proof. $\square$

### Theorem 1

(Correctness of ${\textsc {QX}}$) *Let* $\left\langle {\mathcal {A}},{\mathcal {B}}\right\rangle$ *be a* *p*-*PI. Then,* ${\textsc {QX}}(\left\langle {\mathcal {A}},{\mathcal {B}}\right\rangle )$ *terminates and returns a minimal* *p*-*set wrt.* $\left\langle {\mathcal {A}},{\mathcal {B}}\right\rangle$ *if a* *p*-*set exists for* $\left\langle {\mathcal {A}},{\mathcal {B}}\right\rangle$. *Otherwise,* ${\textsc {QX}}(\left\langle {\mathcal {A}},{\mathcal {B}}\right\rangle )$ *returns ’no* *p*-set’.^20^

### Proof

QX terminates due to Proposition 2.

Proposition 3.(1), first, proves that ’no *p*-set’ is returned if there is no *p*-set wrt. $\left\langle {\mathcal {A}},{\mathcal {B}}\right\rangle$. Second, it shows that, if there is a *p*-set wrt. $\left\langle {\mathcal {A}},{\mathcal {B}}\right\rangle$, ${\textsc {QX}}$ will either return in line 5 or call ${\textsc {QX}}'$ in line 7.

We now show that, in both of these cases, ${\textsc {QX}}$ returns a minimal *p*-set wrt. $\left\langle {\mathcal {A}},{\mathcal {B}}\right\rangle$. This then implies that a minimal *p*-set is returned by ${\textsc {QX}}$ whenever such a one exists.

First, if ${\textsc {QX}}$ returns in line 5, then the output is a minimal *p*-set wrt. $\left\langle {\mathcal {A}},{\mathcal {B}}\right\rangle$ due to Proposition 3.(2).

Second, if ${\textsc {QX}}$-calls ${\textsc {QX}}'({\bar{\varDelta }},\left\langle {\bar{{\mathcal {A}}}},{\bar{{\mathcal {B}}}}\right\rangle )$ in line 7, then $\mathsf {Invar}({\bar{\varDelta }},{\bar{{\mathcal {A}}}},{\bar{{\mathcal {B}}}})$ holds according to Proposition 4. Finally, since $\mathsf {Invar}({\bar{\varDelta }},{\bar{{\mathcal {A}}}},{\bar{{\mathcal {B}}}})$ holds for ${\textsc {QX}}'({\bar{\varDelta }},\left\langle {\bar{{\mathcal {A}}}},{\bar{{\mathcal {B}}}}\right\rangle )$, Proposition 8 establishes that ${\textsc {QX}}'({\bar{\varDelta }},\left\langle {\bar{{\mathcal {A}}}},{\bar{{\mathcal {B}}}}\right\rangle )$ returns a minimal *p*-set wrt. $\left\langle {\mathcal {A}},{\mathcal {B}}\right\rangle$. $\square$

## On the used proof template

To devise the proof presented in Sect. 5, we adhered to a specific proof template. Although several textbooks, e.g., Cormen et al. (2009), Velleman (2006), Edmonds (2008), Kleinberg and Tardos (2006), provide general hints and techniques helpful for proving (recursive) algorithms, we briefly outline the specific template we used in the proof next. The reasons for making this explicit are that knowing the underlying proof template can both promote the understanding of the given proof and serve as a reference point when confronted with the problem of showing the correctness of other recursive procedures.

The foundation for our used template is provided by the proof principle for (non-recursive) algorithms based on loop invariants detailed in Cormen et al. (2009). The idea underlying their framework (which we shall call L-INV) is to prove that a *loop invariant*, i.e., a predicate that is always true while a loop executes, *(1)* holds when the loop is entered (*L-initialization*), *(2)* remains true for the next loop iteration if it is true for the current iteration (*L-maintenance*), and *(3)* yields a property at termination of the loop that is useful to prove the algorithm’s correctness (*L-termination*).^21^ The template (which we will refer to as R-INV) adopted in our proof is an adaptation of L-INV to recursive algorithms. It

- relies on a *recursion invariant*, i.e., a predicate that is true for every recursive call of a procedure, and
- involves the proof that this invariant
  - holds for the first call of the recursive procedure (*R-initialization*),
  - remains true for any further (recursive) call of the procedure
    (*R-maintenance*), and
  - entails correctness of the procedure’s output (*R-termination*).

In spite of the resemblance between R-INV and L-INV, it is essential to understand the different nature of the proof when considering a recursive as opposed to a non-recursive procedure. Whereas R-initialization, similarly as L-initialization, will often be quite easily shown due to its independence from the recursion, addressing R-maintenance and R-termination is more elaborate than L-maintenance and L-termination in general. The reasons are as follows:

1. There is only one entry point into a loop, whereas there may be multiple different places where a recursive procedure can be called. *Consequence:* There is one case to be analyzed for L-maintenance, whereas there can be *multiple cases to be distinguished* for R-maintenance.
2. For a loop, there is no return value and often^22^ only one termination condition, whereas there are multiple termination conditions^23^ for a recursion, and for each such condition a different value can be returned. In particular, a recursive procedure can return due to some trivial case that applies (no nested recursive calls) or after recursively processing a non-trivial case (nested recursive calls). *Consequence:* There is usually one case to be analyzed for L-termination, whereas *multiple cases need to be considered* for R-termination. Moreover, demonstrating R-termination when a non-trivial case is processed by the recursive procedure *requires an induction proof*.
3. In case the recursion implements a divide-and-conquer approach, there can be *combine*-steps where partial solutions are integrated to a complete solution (cf. Alg. 1, line 18). *Consequence:* These *combine-steps need to be addressed* when proving R-termination.

As an illustration, the following table shows how the building blocks of our proof are assigned to the three different proof phases of R-INV:^24^

**Table:** 

**R-initialization** (abbreviations: P...Proposition, T...Theorem)
P4 (invariant holds for first call of ${\textsc {QX}}'$)
**R-maintenance**
*(case 1: recursive* ${\textsc {QX}}'$*-call 1)* P5.(4) (invariant $\Rightarrow$ invariant holds for recursive call in line 16)
*(case 2: recursive* ${\textsc {QX}}'$*-call 2)* P6 (invariant $\Rightarrow$ invariant holds for recursive call in line 17)
**R-termination**
*(correctness of combine-step)* P7 (correct outputs in lines 16, 17 $\Rightarrow$ correct output in line 18)
*(trivial case)* P5.(1) + P5.(3) (invariant $\Rightarrow$ correct output if no nested recursive calls)
*(general case: proof by induction)* P8 (invariant $\Rightarrow$ correct output given nested recursive calls)

Finally, two remarks: *(1)* The finding of a “right” invariant can be tricky and will often represent the key to success in proving a recursive algorithm by means of the R-INV template. However, once an appropriate invariant has been determined, the rest of the proof can be relatively straightforward provided that the systematic steps suggested by R-INV are followed. *(2)* There can be multiple (logically non-equivalent) invariants that allow to prove one and the same algorithm by means of the R-INV template. Indeed, also in the case of QX, a second possible invariant exists and is given by: ${\mathcal {A}}\ne \emptyset \wedge p({\mathcal {A}}\cup {\mathcal {B}})=1 \wedge p({\mathcal {B}}\setminus \varDelta )=0$.^25^

## Conclusion

QuickXplain (${\textsc {QX}}$) is a very popular, highly cited, and frequently employed, adapted, and extended algorithm to solve the MSMP problem, i.e., to find a subset of a given universe such that this subset is irreducible subject to a monotone predicate (e.g., logical consistency). MSMP is an important and common problem and its manifestations occur in a wide range of computer science disciplines. Since ${\textsc {QX}}$ has in practice turned out to be hardly understood by many—experienced academics included—and was published without a proof, we account for that by providing for ${\textsc {QX}}$ an intelligible *proof that explains*. The availability and accessibility of a formal proof is instrumental in various regards. Beside allowing the verification of ${\textsc {QX}}$’s correctness (*proof effect*), it fosters proper and full understanding of ${\textsc {QX}}$ and of other works relying on ${\textsc {QX}}$ (*didactic effect*), it is a necessary foundation for “gapless” correctness proofs of numerous algorithms, e.g., in model-based diagnosis, that rely on (results computed by) ${\textsc {QX}}$ (*completeness effect*), it makes the intuition of ${\textsc {QX}}$’s correctness bullet-proof and excludes the later detection of algorithmic errors, as was already experienced even for seminal works in the past (*trust and sustainability effect*), as well as it might be used as a template for devising proofs of other recursive algorithms (*transfer effect*). Since *(i)* we exemplify the workings of ${\textsc {QX}}$ using a novel tried and tested well-comprehensible notation, and *(ii)* we put a special emphasis on the clarity and didactic value of the given proof (e.g., by segmenting the proof into small, intuitive, and easily-digestible chunks, by showing how our proof can be “directly traced” using the recursive call tree produced by ${\textsc {QX}}$, and by explaining the underlying proof template with the intention to make it reusable for other proofs), we believe that this work can decisively contribute to a better understanding of ${\textsc {QX}}$, which we expect to be of great value for both practitioners and researchers.

## Data Availability

Not applicable.

## Footnotes

1 Judged by taking the citation tally on Google Scholar as a criterion; as of October 2021, the QuickXplain paper boasts 510 citations.

2 In many manifestations of the MSMP problem, predicate decision procedures are implemented by theorem provers, e.g., SAT-solvers (Marques-Silva et al. 2013) or description logic reasoners (Rodler 2015).

3 In our research and teaching on model-based diagnosis, we frequently discuss and analyze ${\textsc {QX}}$—one of the core algorithms used in our works and prototypes—with students as well as other faculty (including highly proficient university professors specialized in, e.g., algorithms and data structures). The feedback of people is usually that they cannot fully grasp the workings of ${\textsc {QX}}$ before they take significant time to go through a particular example thoroughly and noting down all single steps of the algorithm. According to people’s comments, the main obstacle appears to be the recursive nature of the algorithm. A recent structured survey among computer science researchers and university teachers in this regard made these experiences even more explicit and concrete (the results of the survey can be accessed at http://isbi.aau.at/ontodebug/evaluation).

4 A prominent example which shows that even seminal papers are not charmed against errors in absence of formal proofs, and thus underscores the importance of (public) proofs, is the highly influential paper of Raymond Reiter from 1987 (Reiter 1987). It proposes the hitting set algorithm for model-based diagnosis, but omits a formal proof of correctness. And, indeed, a critical error in the algorithm was later found (and corrected) by Greiner et al. (1989).

5 The original algorithm was depicted specifically as a searcher for explanations or relaxations for over-constrained constraint satisfaction problems (CSPs). Although the proper interpretation of the original formulation to address arbitrary MSMP problems different from CSPs may be relatively straightforward (for people familiar with CSPs), we believe that our more general depiction (cf. Marques-Silva et al. (2013)) can help readers non-familiar with the domain of CSPs to understand and correctly use ${\textsc {QX}}$ without needing to properly re-interpret concepts from an unknown field.

6 We (informally) experimented with different variants how to explain ${\textsc {QX}}$, and found out (through the feedback of discussion partners, e.g., students) that the shown representation was more accessible than others.

7 Throughout this paper, minimality always refers to minimality wrt. set-inclusion.

8 Cf. (2) with Proposition 5 (unproven) in Junker (2004).

9 The analyzed set, denoted by ${\mathcal {A}}$ in Alg. 1, is referred to by ${\mathcal {C}}$ in the original algorithm (Junker 2004).

10 Clearly, the checks in lines 9 and 11 are executed in every recursive ${\textsc {QX}}'$-call. However, in all further recursive calls, these tests serve to determine whether a particular given *subset* of the analyzed set must be further processed or not. We separately discuss the first two such checks *related to the original input problem* in this paragraph in order to cover *all* trivial cases before moving on to elucidate the recursion.

11 Remarks: *(1)* $\varDelta = {\mathcal {B}}$ in the very first execution of ${\textsc {QX}}'$, cf. lines 7 and 8. *(2)* $p({\mathcal {B}})=1$ implies that the first condition $\varDelta \ne \emptyset$ checked in line 9 is true as well (cf. Definition 1). *(3)* Actually, the check in line 9 in the very first execution of ${\textsc {QX}}'$ complements the one in line 4. The reason is that in line 4 ${\mathcal {A}}= \emptyset$ and $p({\mathcal {B}})=1$ (due to line 2) holds, whereas in line 9 ${\mathcal {A}}\ne \emptyset$ and $p({\mathcal {B}})=1$ is true.

12 If ${\textsc {QX}}'$ was called in line 7, the truth of the condition tested in line 9 depends only on $p({\mathcal {B}})$ (cf. Footnote 11) and thus $\varDelta$ has no effect. Similarly, given that ${\textsc {QX}}'$ was called in line 16, $\varDelta$ always corresponds to the non-empty set ${\mathcal {A}}_1$ and, again, $p({\mathcal {B}})$ alone determines the truth value of the condition in line 9.

13 Such an element is commonly referred to as a *necessary* or a *transition* element (Belov and Marques-Silva 2012).

14 We intentionally abstain from a notation which is guided by the call-recursion-tree or which lists all variables and their values (which we found was often perceived difficult to understand, e.g., since same variable names are differently assigned in all the recursive calls). The reason is: While explaining ${\textsc {QX}}$ to people (mostly computer scientists) using various representations, we found out via people’s feedback that the presented “flat” notation could best convey the intuition behind ${\textsc {QX}}$; moreover, it enabled people to correctly solve new examples on their own.

15 An example of a different notation that describes the workings of ${\textsc {QX}}$ based on the call-recursion-tree can be found, e.g., in Fig. 4.1 in Rodler (2015).

16 In the proof, we will often talk about different calls of ${\textsc {QX}}'(\varDelta ,\left\langle {\mathcal {A}},{\mathcal {B}}\right\rangle )$ while ${\textsc {QX}}(\left\langle {\mathcal {A}},{\mathcal {B}}\right\rangle )$ executes. To account for the facts that *(a)* the actual parameters passed to ${\textsc {QX}}'$ will generally differ at each call, and *(b)* the actual parameters passed to ${\textsc {QX}}'$ will generally not be equal to the original ${\mathcal {A}},{\mathcal {B}}$ (passed to ${\textsc {QX}}$), we (have to) use different designators $X',{\bar{X}},{\dot{X}}$ and ${\ddot{X}}$ for these parameters $X \in \{\varDelta ,{\mathcal {A}},{\mathcal {B}}\}$ for each discussed ${\textsc {QX}}'$-call.

17 Cf. Theorem 1 (unproven) in Junker (2004).

18 Cf. Proposition 6.2 (unproven) in Junker (2004).

19 That is, *additional* calls made, *not* taking into account the running routine ${\textsc {QX}}'({\bar{\varDelta }},\left\langle {\bar{{\mathcal {A}}}},{\bar{{\mathcal {B}}}}\right\rangle )$ that we consider in the proposition.

20 Cf. Theorem 1 in Junker (2004).

21 The three stages of the proof are originally called initialization, maintenance and termination in Cormen et al. (2009); we added the L-prefix to distinguish these terms relating to loops from the newly introduced ones, which are associated with recursion (R-prefix).

22 If break-statements do not occur within the loop.

23 One such “condition” is reaching the last statement of the procedure.

24 To be precise, the table shows the steps of the proof that ${\textsc {QX}}'$, i.e., the recursive part of ${\textsc {QX}}$, is correct. To show that ${\textsc {QX}}$ is correct, Theorem 1 roughly combines the correctness of the trivial cases (checked before ${\textsc {QX}}'$ is called) with the correctness of ${\textsc {QX}}'$ (cf. Fig. 2).

25 We thank a researcher colleague for making us aware of this alternative invariant for proving QX.
